# Conflicting and complementary notions of responsibility in caregiver’s and health care workers’ vaccination narratives in the Philippines

**DOI:** 10.7189/jogh.14.04016

**Published:** 2024-01-12

**Authors:** Ma Leslie Ulmido, Mark Donald C Reñosa, Jonas Wachinger, Vivienne Endoma, Jhoys Landicho-Guevarra, Jeniffer Landicho, Thea Andrea Bravo, Mila Aligato, Shannon A McMahon

**Affiliations:** 1 Heidelberg Institute of Global Health, Ruprecht-Karls Universität Heidelberg, Heidelberg, Baden-Württemberg, Germany; 2 Department of Epidemiology and Biostatistics, Research Institute for Tropical Medicine - Department of Health, Muntinlupa City, Metro Manila, Philippines; 3 International Health Department, Johns Hopkins University, Bloomberg School of Public Health, Baltimore, Maryland, USA

## Abstract

**Background:**

Vaccine hesitancy (VH) continues to pose a public health threat globally. Understanding the attitudes and perceptions about vaccination of key stakeholders in vaccine decision-making (such as health care workers (HCWs) and caregivers) about vaccination can pave the way toward novel approaches to bolster vaccine confidence. In this study, we explored the role of notions of responsibilities among HCWs and caregivers in shaping vaccination interactions and decision-making in the Philippines.

**Methods:**

We conducted in-depth interviews (IDIs) and focus group discussions (FGDs) with 44 vaccine-hesitant caregivers, seven HCWs, and 20 community health workers (barangay health workers) in the Philippines between August 2020 and March 2021. The interviews and focus groups were conducted online, transcribed verbatim, and analysed through the reflexive thematic analysis approach.

**Results:**

Caregivers highlighted responsibility in terms of being a good caregiver, managing risk to one’s own child, and seeking and validating information. Meanwhile, HCWs highlighted responsibility as: being a good HCW, managing risk to children and to the community, and providing and transforming information. Our findings suggest that responsibility manifests differently in HCWs’ and caregivers’ narratives, and that these notions can be both conflicting and complementary, shaping the interaction between stakeholders and, ultimately, their vaccine decision-making. We also found that fostering a good relationship between HCWs and caregivers through communication techniques such as motivational interviewing could help bridge the gap created by mistrust in vaccinations. HCWs sharing their own experiences as parents who vaccinate their own children also resonate with caregivers.

**Conclusions:**

Notions of responsibility can underpin collaborative and divisive interactions between HCWs and caregivers. Public health messaging and interventions related to vaccination must consider strategies that align with these notions to address VH.

Vaccines are essential in the global fight against infectious diseases, as they are cost-effective and safe for preventing millions of deaths annually, particularly among young children [1]. However, recent declines in vaccination rates, often associated with increased vaccine hesitancy (VH), threaten decades of progress in reducing child mortality and morbidity. This decline has resulted in outbreaks of previously controlled or eradicated vaccine-preventable diseases (VPDs) in several countries [2]. Consequently, VH was identified among the top 10 global public health threats affecting both individuals and communities [3,4].

Low- and middle-income countries (LMICs) are particularly vulnerable to the threats of VPDs due to the frequently insufficient infrastructure and supply chain inefficiencies of their health systems [5]. Currently, most of the VPDs are recorded in LMICs, which places young children at significant risk from morbidity and mortality [5,6]. One such LMIC is the Philippines, which has recently experienced an increase in VH; once among the top 10 countries globally in terms of vaccine confidence in 2015, it had dropped to the 70^th^ place by 2019 [2]. Furthermore, a 2023 UNICEF report highlighted that the perception of the importance of childhood vaccines declined by about 25% in the Philippines during the coronavirus disease 2019 (COVID-19) pandemic [7]. The 2016 Dengvaxia controversy, which involved the abrupt withdrawal of a novel dengue vaccine, further exacerbated the drop of vaccine confidence [8,9]. Since then, caregivers have remained ambivalent (and in many cases hesitant) about vaccination, while HCWs have been attempting to restore their trust in the longstanding childhood vaccination program [10].

Research suggests that understanding HCWs’ and caregivers’ attitudes and perceptions about vaccination can pave the way toward increasing vaccine confidence, as these two groups play a substantial role in vaccine decision-making [11–14]. However, less attention has been given to different aspects of vaccination that are closer to social sciences, such as motivations for vaccination and notions of responsibility.

For caregivers, especially for parents of young children, social prescriptions subject them to expectations to construct themselves as ‘responsible citizens’ by caring for their children and devoting a significant amount of time and attention to learning the best parenting practices in their child’s best interests [15,16]. Thus, the responsibility of maintaining children’s health and protecting them against sickness and harm falls upon caregivers [17]. However, vaccination can be seen as a paradox among caregivers, as it often includes risks and pain that goes against their intrinsic motivation to avoid harming their children’s ‘vulnerable bodies,’ while public health authorities often rationalise that responsible caregivers vaccinate their child [17–19]. In contrast, HCWs involved in vaccination programs have different views on vaccination due to their training, their backgrounds, and their role in ensuring the child’s and the community’s health [20]. However, due to the boundaries of ‘consent’ and ‘free choice,’ HCWs need to work with caregivers who act as ‘gatekeepers’ of vaccination [20]. In both cases, ‘responsibilities’ among caregivers and HCWs in line with vaccination stem from duties and obligations delegated to them based on their role in society. Thus, understanding the different notions of responsibilities of HCWs and caregivers in terms of vaccination can pave the way to understanding how these notions influenced their interactions and decision about vaccines.

To address gaps in the literature, we aimed to explore HCWs’ and caregivers’ perceptions of responsibility regarding vaccination for children under five in the Philippines.

## METHODS

### Design, setting, and participants

This study is part of a larger mixed methods study set in the Philippines, to develop a vaccine-confidence intervention based on human-centred design [21]. Detailed study procedures have been published elsewhere [22].

We conducted our study in the Calabarzon region, which reported a three-fold surge in measles cases in 2019 as compared to the preceding year [23–25]. Based on the regional Expanded Programme on Immunization 2018-2019 coverage reports, we purposively selected one district in Dasmariñas City and one municipality in Cavite Province, which have the lowest vaccination rates among children under five. The chosen settings included both rural and urban conditions to allow for recruiting participants from different socio-demographic backgrounds, religious groups, and family household structures, and differing access to health facilities.

The study population included caregivers of children under five and HCWs (including medical doctors, nurses, midwives, and community health workers (barangay health workers (BHWs) in this context)). With the help of BHWs, we purposively selected caregivers who had delayed or refused at least one childhood vaccine between 2018 and the date of data collection. We included HCWs employed under the municipal health office (MHO) of Cavite province who managed and implemented the vaccination programs and were residents of the study setting.

### Data collection

Following official communication with the Philippines Department of Health and local officials and securing written informed consent, five qualified and trained interviewers (VE, JLG, JL, TAB, and MA) collected qualitative data using standardised in-depth interviews (IDI) and focus group discussions (FGD) guidelines. All interviewers were Filipino nationals, female, and held bachelor’s degrees in nursing, midwifery, or the social sciences. They had between two and 15 years of experience in qualitative research. They were fluent in both Filipino and English, allowing them to adapt to the language preferences of the participants. All interviewers participated in a study-specific five-day training on qualitative data collection. This training covered topics such as modules on VH, different interviewing techniques, research ethics, software resources, and strategies to build rapport during online interviews.

IDIs were conducted to elicit nuanced and in-depth experiences and narratives from respondents. For caregivers, the IDIs focus on their experiences in childrearing and in accessing health services for their child, in particular, vaccination services. The IDIs with HCWs were aimed at understanding their experiences related to childhood vaccination in the communities and health facilities at which they were employed. Lastly, we also conducted four unstratified FGDs with BHWs to understand their experiences in providing health services to the communities, as well as social norms and prescriptions surrounding childhood vaccination.

We adopted remote data collection tools and online platforms to adhere to governmental regulations due to COVID-19 restrictions [26]. Interviews were scheduled according to the participant’s preference, and they were asked to choose a private and comfortable location to ensure privacy. Due to the online nature of the interviews, the interviewers could not always guarantee that no other individuals came into hearing distance for the entire session. If disturbances by others were noticed by the interviewers, they allowed respondents to decide whether to continue, halt, or reschedule the interview. No respondent opted to reschedule or expressed privacy concerns.

We collected data between August 2020 and March 2021. Interviews and FGDs were semi-structured using open-ended questioning techniques.

### Data analysis

The interviews, whether in audio or video format, were transcribed verbatim (in Filipino) and then translated into English by trained data transcribers who were proficient in both languages. The transcription process followed qualitative standards to ensure accuracy and consistency [27]. We used NVivo, version 12.7.0 (3873) (QSR International, Burlington, MA, USA) to organise and code data. Data analysis took place from February 2022 until June 2022. Reflexive thematic analysis (RTA) guided our analytic approach, allowing the identification and reporting of recurring patterns or themes in the collected data [28].

The lead author (MLU) initially performed deductive coding guided by Shalom Schwartz’ Norms Activation Theory (NAT), which outlines the relationship between notions of responsibility and personal and social norms [29]. However, not all themes that emerged within the coded data were fully reflected in the NAT, so MLU revisited the initial codes and restructured them inductively. Hence, we adopted both a deductive (based on the initial NAT) and inductive coding process (based on new codes that emerged from the data).

The linkages and relationships between the various themes were further analyzed, leading to the development of a framework titled ‘Conflicting and complementary notions of responsibility of health care workers and caregivers on childhood vaccination.’

### Ethics and ethical implications

The Institutional Review Board of the Research Institute for Tropical Medicine, Philippines (No. 2019-44) and the Ethical Commission of Heidelberg University, Faculty of Medicine (S-833/2019) provided ethical approval for this study. We adhered to the principles of ethical research on human subjects outlined in the Declaration of Helsinki [30].

## RESULTS

We approached 55 caregivers of children under five who had previously delayed or refused vaccination and completed 44 interviews. Reasons of non-participation were busy schedules (n = 2) and the husband or other family members refusing to participate (n = 4); five individuals cited no specific reasons for their refusal. The interviews with caregivers lasted 63 minutes (range = 30–105) on average. Most participating caregivers were female (n = 42), <30 years old on average, and high school graduates. Meanwhile, most of the HCWs were female, <50 years old, and with >10 years of service. Interviews with HCWs took an average of 66 minutes (range = 42–80).

The FGDs with BHWs lasted 94 minutes (range = 75–134) on average. All BHWs participating in the FGDs were female, aged 30 to 50 years (median age of 46.5 years), and had spent fewer years in service on average compared to the interviewed HCWs. The longest years in service recorded was 25 years. However, we had missing data on the number of years in service for one participant (**Table 1**).

**Table 1:** Demographic profiles of interview participants, presented as n (%)

	Caregivers, n = 44	Health care workers, n = 7	Barangay health workers, n = 20*
**Sex**			
Male	2 (4.55)	1 (14.29)	0 (0)
Female	42 (95.45)	6 (85.71)	20 (100)
**Age group**			
<30	17 (38.64)		
30–40	16 (36.36)	1 (14.29)	7 (35)
41–50	8 (18.18)	3 (42.86)	8 (40)
51–60	3 (6.82)	3 (42.86)	5 (25)
**Number of children**			
1–2	15 (34.09)	-	-
3–4	16 (36.36)	-	-
5–6	8 (18.18)	-	-
7–8	2 (4.55)	-	-
9–10	2 (4.55)	-	-
>10	1 (2.27)	-	-
**Educational attainment**			
None	2 (4.55)	-	-
Primary	10 (22.73)	-	-
High School	24 (54.55)	-	-
College	7 (15.91)	-	-
Vocational training	1 (2.27)	-	-
**Cadre**			
Medical doctor	-	2 (28.57)	-
Registered nurse	-	2 (28.57)	-
Registered midwife	-	3 (42.86)	-
**Number of years in service**			
0–1	-	-	2 (10.53)
<10	-	3 (42.86)	13 (68.42)
11–20	-	1 (14.29)	3 (15.79)
21–30	-	2 (28.57)	1 (5.26)
>30	-	1 (14.29)	-

*Focus group discussion participants. One participant with missing data.

### Framework on conflicting and complementary notions of responsibility

**Figure 1:**
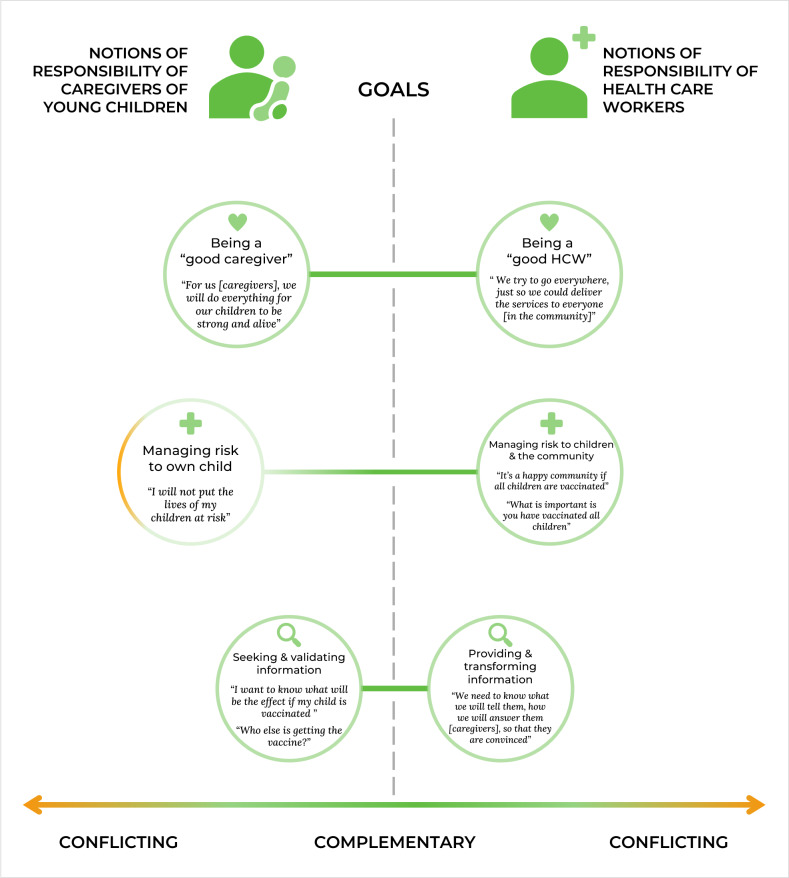
Conflicting and complementary notions of responsibility of health care workers and caregivers on childhood vaccination. Each circle represents the notions of responsibility identified in their narratives. The left side of the figure illustrates the notions of responsibility of caregivers, including being a good caregiver, managing risk to one’s own child, and seeking and validating information. The narratives from HCWs, meanwhile, reveal several notions of responsibility, such as being a good HCW, managing risk to children and the community, and providing and transforming information. The scale below visualises how each notion of responsibility is positioned relative to that of the other participant (e.g. being a good caregiver *vis-à-vis* being a good HCW). The middle line represents a meeting point where both participant groups’ responses demonstrate complementarity. The greater distance between each circle moves from the middle line signifies more conflicting notions of responsibility.

We present our findings within a framework entitled ‘Conflicting and complementary notions of responsibility of HCWs and caregivers on childhood vaccination’ to facilitate understanding of the varying notions of responsibility held by caregivers and HCWs (**Figure 1**).

### Caregiver’s notions of responsibility

#### Being a good caregiver

Respondents conveyed being a good caregiver as a responsibility that entailed putting the needs of the child and family first. In this vein, they relayed their willingness to make sacrifices or compromises and overcome difficulties to ensure their child’s health. For example, despite voicing financial challenges, respondents shared a willingness to sacrifice financial resources to secure vaccines for their child. However, several respondents saw vaccines not as a requirement or a necessity, but as an option for good caregiving. For example, in lieu of vaccinations, caregivers emphasised other practices to ensure the health of their children, including proper nutrition, practicing breastfeeding, and watching over their children to account for risks and exposures that could be harmful, such as ‘preventing them from going out when it is cold to avoid cough or colds’ (mother of three, 25 years old).

Respondents also expressed experiencing several, sometimes conflicting responsibilities, resulting in situations where they had to prioritise which one to fulfil. For instance, a mother expressed that being a good caregiver also required her to sometimes prioritise daily parenting responsibilities over her children’s vaccination: ‘I am alone because my partner works far away, I need to go to the market, and cook so others can eat, I finish my chores so late sometimes’ (mother of six, 33 years old). Caregivers also explained how social pressure, expectations, and peer judgment added tension to their already demanding caregiving roles. Respondents emphasised how Filipino societies had a socially constructed view of ‘good caregivers’ as someone who allows vaccination for their children to protect them from childhood illnesses. For example, a caregiver who had chosen to vaccinate their children remarked that caregivers who fail to do so are ‘neglectful’ or ‘careless’ (grandmother, 50 years old).

#### Managing risk to one’s own child

Respondents felt responsible in managing risks for their children – which could include vaccinating when felt that it would reduce the risk for their child to contract diseases, but could also result in vaccine refusal if the perception emerged that vaccines themselves were too risky. Caregivers also mentioned being cautious during the vaccination process to mitigate risks. For example, when probed on their strategies to guarantee vaccine safety, caregivers shared how they stayed ‘observant’ during the entire procedure and would ‘not take away my eyes from the needle, and I always watch during the vaccination session’ (mother of five, 30 years old). Another caregiver said that she only accepted vaccines from ‘HCWs I know and refused those given by other HCWs whom I am not acquainted with’ (grandmother, 52 years old).

In line with this iterative assessment of risks, caregivers emphasised that an acceptance of one vaccine did not guarantee their general consent to all vaccines. Similarly, a refusal of one vaccine did not automatically rule out other future vaccinations. Caregivers spoke extensively about the need for vaccines included in the routine vaccination program, highlighting in conversations that the ‘old’ vaccines are safer (mother of two, 31 years). Conversely, most caregivers expressed particular concern regarding ‘new vaccines’ introduced by the Department of Health and those newly out in the country (mother of two, 21 years old and mother of two, 22 years old). For instance, a caregiver expressed her fear of accepting new vaccines due to the Dengvaxia controversy: ‘I am scared to vaccinate my kids like with Dengvaxia, I will not vaccinate, at least (not) until others have tried it, if they tried it and nothing happened, and the results are good, I can inject’ (mother of nine, 43 years old).

#### Seeking and validating information

Caregivers also saw it as their responsibility to actively seek out and validate information on vaccination, which included having an active role in deciphering confusing information from various sources such as family members, friends, or other community members. Upon probing, caregivers reported that HCWs remained their most trusted source of information due to their role in promoting child and community health. They mentioned that HCWs are ‘professionals’ and the ones who are ‘experts in vaccination’ (mother of five, 30 years, and father of three, 46 years, respectively). However, caregivers expressed their desire to obtain more information regarding the vaccine safety, benefits, and associated risks.

### Health care workers’ notions of responsibility

#### Being a good HCW

Being a good HCW emerged as a responsibility among HCWs, which included ensuring that their work tasks were achieved despite challenges and delivering health services by partnering with community members (e.g. caregivers). However, they did note that vaccination was only one of their many tasks, and that keeping track and balancing multiple responsibilities is challenging (community health nurse, 59 years old). Despite their heavy workload and inadequate compensation, HCWs reported feeling satisfied with helping members of the community (BHW, 51 years old). BHWs also cited that they treat children in the community as if ‘they were their own,’ highlighting their dedication to caring for them. HCWs further expressed that finally convincing vaccine hesitant caregivers through their effort to constantly follow-up and visit them was very rewarding (midwife, 58 years old). For them, ‘it is a happy community when all children are vaccinated’ (midwife, 44 years old).

#### Managing risk to children and to the community

Preventing outbreaks, vaccinating all children, and achieving broad goals set by health authorities were all part of managing risk to children, both individually and collectively. HCWs relayed that unmanaged outbreaks might add extra levels of work not just to themselves, but to the whole health system, and that they would be the ones to ‘suffer’ consequently (national immunisation program coordinator, 45 years old). HCWs expressed a sense of duty to steer caregivers in the direction of vaccination, resorting to various strategies to entice them, such as house-to-house visits and providing medicines or vitamins. For some respondents, this also included asking the help of selected caregivers to share vaccine information with others.

However, HCWs expressed how they sometimes had to resort to verbal intimidation and filing complaints to local authorities while dealing with vaccine hesitant caregivers and reach vaccination targets. For instance, a midwife recalled how their doctor threatened caregivers to stop health services in the community if they would not vaccinate: ‘If you are all like that, I will stop everything, including free circumcision, you can’t have family planning, no more prenatal with (name), stop everything’ (midwife, 58 years old).

#### Providing and transforming information

Being a responsible HCW included the provision and transformation of information. According to respondents, this entailed being able to provide information that would be easily understood by caregivers in the communities. During vaccination, HCWs informed caregivers of the possible vaccine-associated side effects and how to manage them. However, due to multiple workloads, respondents often felt they lacked time to address all questions and concerns of caregivers. BHWs also expressed that they lacked knowledge on vaccination or felt hesitant to answer some of the caregivers’ questions, citing that they were not ‘clinically trained’ or not a ‘medical professional’ (BHW, 47 years old). They shared general information with mothers, such as ‘vaccines are good for your kids’ or ‘vaccines help the kids to be healthy and prevent them from getting sick.’

### Conflicting and complementing notions of responsibility between HCWs and caregivers

Being a good caregiver and being a good HCW often emerged as complementing responsibilities. Part of being a good caregiver includes wanting to achieve good health and well-being of the child, which entails the aid of HCWs. Likewise, HCWs’ accounts showed that they also work for and with caregivers to promote child health. However, conflict may also arise due to many self-described good caregivers noting that vaccination can be considered an option based on parental determination of risk, while HCWs see vaccination as a ‘parental responsibility’ (BHW, 47 years old). With multiple responsibilities in the family unit, vaccination can also fall behind other caregivers’ priorities.

Seeking information and providing information were also complementary notions of responsibility of caregivers and HCWs, respectively. Specifically, caregivers seek and validate information, while HCWs provide and transform information. HCWs showed awareness of the nature of information that caregivers need to help them in vaccine decision-making. However, caregivers still reported a perceived lack of information, noting that HCWs were prone to providing general information that could not fully address their concerns. Additionally, insights from neighbours, friends, and online discourse were messages that contradicted HCW messaging.

Finally, tension exists between the competing notions of responsibility in managing risk to one’s own child and managing risk to children and to the community. While caregivers make vaccine decisions on an individual basis based on their assessment of risk to their child, HCWs view vaccination as an intervention that is ‘fit for all children’ in the community. Thus, HCWs were perceived as disregarding the concerns of caregivers, and HCWs themselves provided examples that would underpin this. For instance, when mothers mentioned being afraid that their child will have a fever after vaccination, one BHW regarded this as ‘stubbornness’ and mentioned that ‘the health center will provide free medicines, that’s just it’ (BHW, 60 years old). Despite tensions, these notions of responsibility could also align. For example, a mother allowed polio vaccination for her child as she shared that ‘I don’t know of any way to prevent polio aside from the injection’ (mother of two, 23 years old).

## DISCUSSION

Our findings highlight how HCWs and caregivers perceive their responsibilities regarding childhood vaccination. Notions of responsibility emerging from the accounts of caregivers include being a good caregiver, managing risk to one’s own child, and seeking and validating information. Meanwhile, HCWs perceive being a good HCW, managing risk to children and to the community, and providing and transforming information as core responsibilities. The results suggest that these notions could be conflicting and complementary, thereby affecting the interaction between the two groups and, ultimately, their vaccine decision-making.

In our study, notions of responsibility of caregivers focussed on his or her child and family, with the primary goal of ensuring the child’s health and wellness. This aligns with previous studies in which some caregivers regarded vaccines as part of their responsibility, while others perceived vaccines as an option for maintaining their child’s health [16,31–33]. However, a study conducted in eight countries in Europe and Asia suggested that vaccines are considered a ‘socially forced choice,’ thus putting additional pressure to parents, as they are acting as ‘proxy decision makers for their children, who are unable to decide for themselves’ [34]. In this context, caregivers’ vaccination choice could be greatly influenced by their setting, despite having the notion that vaccine is an ‘option’. Similarly, caregivers in our study who refused vaccination were seen as ‘careless’ and ‘neglectful’ by other caregivers for their vaccine choices, emphasising the social construct that a ‘good caregiver’ vaccinates.

Our findings also highlight that vaccine decision-making is heavily influenced by caregivers’ perceptions of vaccine risks, which discourages them from vaccinating their children despite societal pressure and public health messaging to do so. The spread of misinformation and rumors about vaccines also makes it difficult to settle on a course of action [35,36]. Caregivers, seeing it as their responsibility to minimize their children’s exposure to harm, turn to a variety of vaccine strategies to aid them in making informed decisions about vaccination [16,20,31,33]. However, these personalized vaccine strategies, such as devising their own vaccination schedules, delaying consent, or selecting vaccination, may be seen as “irrational” from the public health perspective [20].

Caregivers also felt responsible for seeking and validating information to help them in vaccine decision making. A study in the USA noted that caregivers conduct their own ‘research’ to learn about the risks and benefits of vaccination, which includes consulting books, searching the internet, watching documentaries, and listening to their significant others, such as friends and family [20]. However, due to the various sources of information, caregivers opined that they were left confused when navigating vaccination information [16]. Caregivers in various study settings shared that they obtain information from their trusted source as a way to counter these rumours and misinformation-the HCWs [37,38]. This resembles our findings that caregivers chose HCWs as their main source of vaccine information amid the proliferation of misinformation and conspiracy theories.

Conversely, HCWs in our study felt responsible for the health of the community as a whole and not only the needs of an individual child. Their notions of responsibility coincide with their role as ‘overseers’ of the community's health [20]. They tend to focus on achieving their tasks-convincing caregivers to accept vaccination, in the purview of meeting their targets and preventing outbreaks in the community as they are accountable to their supervising officers. This concurs with a study in India, where HCWs were blamed when levels of immunisation coverage failed to meet the targets [39].

HCWs in our study felt responsible for providing and transforming information for caregivers. Similarly, those in Ireland, South Africa, Nigeria, and Australia regarded providing information as a responsibility rooted in their roles as health care providers [40–43]. However, HCWs are sometimes unresponsive to the needs of caregivers for vaccine information or regard themselves as having inadequate knowledge to guide caregivers [40,42]. For example, a study in Nigeria found health facilities to be understaffed and HCWs overburdened, resulting in them not being able to address the need of the caregivers for vaccine information [41].

As highlighted in this study, the conflicting nature of caregivers’ and HCWs’ notions of responsibilities has the potential to instigate tensions and mistrust in their interactions. To bridge this gap, HCWs can reach out and partner with caregivers to enable good vaccination decisions despite the latter’s hesitations and risk perceptions. Appealing to the caregiver’s instincts, HCWs in the USA and Ireland have found that an effective communication strategy to convince VH caregivers involves referencing their own personal experiences and choices as parents [43,44]. These findings highlight potential overlaps in the notions of responsibility of HCWs and caregivers in managing risk to a child. Kempe et al. [44] highlighted that, by sharing their personal vaccine decisions, HCWs emphasise the safety and effectiveness of vaccines, removing professional barriers and acting as fellow parent who wants ‘the best care for their child.’

Instead of resorting to verbal coercion to force caregivers to vaccinate which was described among our study respondents, HCWs could build a trustful patient-provider relationship to increase confidence in vaccination through positive interaction [45]. This can be done by having HCWs listen respectfully to parental concerns, encouraging questions, and providing accurate information about the risks and benefits of vaccines which aligns with the notions of responsibilities of caregivers [46]. For instance, motivational interviewing techniques were found to be effective in establishing trustful patient-provider interaction and facilitating vaccine acceptance in various settings [47–50].

Addressing health systems challenges is also crucial in facilitating change in communication and interaction between HCWs and caregivers. Despite the notions of responsibilities of HCWs to be good HCWs by performing their duties and promoting community and child health, they often face challenges due to the multiple vertical programmes that they handle in the communities. Likewise, a study by Decouttere et al. [51] in Rwanda showed that HCWs’ multiple tasks could contribute to the poor quality of immunisation services due to insufficient manpower to operate the facility and to serve the members of the community. This echoes our findings where caregivers perceived that the HCWs were unable to address caregivers’ worries and fears, resulting in a perceived low quality of services. Therefore, besides trying to foster good relationships between HCWs and caregivers, policymakers should also strive to address health system challenges, especially in LMICs, to motivate HCWs and to entice caregivers to seek care from public health facilities [52].

### Limitations

The study’s limitations include self-report bias, which could have introduced social desirability and recall bias among the participants, and a shift to online data collection, which may have excluded some participants who lacked the necessary devices. Further, most of our participants were mothers, limiting our insights into the caregiving practices and perceptions of other family members, such as fathers.

## CONCLUSIONS

Our findings outline how the notions of responsibility can underpin collaborative and divisive interactions between HCWs and caregivers, and therefore merit consideration when crafting public health messaging and developing interventions related to vaccination. Caregivers want to feel that their concerns are recognised and that their decisions are not rushed while HCWs remain their trusted source of information. As such, partnering and fostering good relationships between caregivers and HCWs through aligning with their notions of responsibility is needed to facilitate vaccination uptake and improve their vaccine interaction. Finally, we urge more research on the topic of responsibility and how this could be better leveraged to address VH in the Philippines and globally.
